# Molecular Dynamics Study on Mechanical Properties of Nanopolycrystalline Cu–Sn Alloy

**DOI:** 10.3390/ma14247782

**Published:** 2021-12-16

**Authors:** Guodong Zhang, Junsheng Zhao, Pengfei Wang, Xiaoyu Li, Yudong Liu, Xinyue Fu

**Affiliations:** School of Mechanical Engineering, North University of China, Taiyuan 030051, China; zgd96026@163.com (G.Z.); wangpf20210001@nuc.edu.cn (P.W.); yuyu963210@126.com (X.L.); liuyudong00@163.com (Y.L.); xiaoyue__996@163.com (X.F.)

**Keywords:** molecular dynamics, Cu–Sn alloy, nanopolycrystalline, Sn content, strain rate

## Abstract

Molecular dynamics simulation is one kinds of important methods to research the nanocrystalline materials which is difficult to be studied through experimental characterization. In order to study the effects of Sn content and strain rate on the mechanical properties of nanopolycrystalline Cu–Sn alloy, the tensile simulation of nanopolycrystalline Cu–Sn alloy was carried out by molecular dynamics in the present study. The results demonstrate that the addition of Sn reduces the ductility of Cu–Sn alloy. However, the elastic modulus and tensile strength of Cu–Sn alloy are improved with increasing the Sn content initially, but they will be reduced when the Sn content exceeds 4% and 8%, respectively. Then, strain rate ranges from 1 × 10^9^ s^−1^ to 5 × 10^9^ s^−1^ were applied to the Cu–7Sn alloy, the results show that the strain rate influence elastic modulus of nanopolycrystalline Cu–7Sn alloy weakly, but the tensile strength and ductility enhance obviously with increasing the strain rate. Finally, the microstructure evolution of nanopolycrystalline Cu–Sn alloy during the whole tensile process was studied. It is found that the dislocation density in the Cu–Sn alloy reduces with increasing the Sn content. However, high strain rate leads to stacking faults more easily to generate and high dislocation density in the Cu–7Sn alloy.

## 1. Introduction

Cu–Sn alloy is one kind of traditional copper alloy, which has high hardness, strength, wear resistance, corrosion resistance and good ductility and is widely used in mechanical manufacturing, such as wear-resistant, corrosion-resistant, high-strength load bearing parts [1,2,3]. Due to the reliability demand of bearing parts, which require excellent comprehensive mechanical properties, it is valuable and practical to research the influencing factors of strength, ductility and elastic modulus for Cu–Sn alloy. Although there have been a large number of studies of Cu–Sn alloy [4,5,6], the research on the mechanical properties of nanopolycrystalline Cu–Sn alloy at the atomic level is limited.

In recent decades, the alloys with nano-scale grains have attracted lots of researchers due to their excellent exceptional mechanical properties. With the rapid development of material technology, and the further improvement of the requirements of engineering application for material properties, nanocrystalline materials have attracted the increasing attention of many researchers [7,8]. However, nanopolycrystalline Cu–Sn alloys are seldom studied.

Moreover, due to the size limitation of nanopolycrystalline materials and the complexity of experimental conditions, it is very difficult to research nanopolycrystalline Cu–Sn alloys. Therefore, molecular dynamics simulation has become a useful tool to study the mechanical properties of nanopolycrystalline materials. Numerous researchers have found that the macro mechanical properties and deformation behavior of materials are closely related to many factors, among which the element ratio [9,10,11,12] and strain rate [13,14,15,16] more significantly affect the mechanical properties of the alloy system. Zhao [17] et al. studied the effect of Pb content on the mechanical properties of Cu–Pb alloy. They found that with the increase of Pb content the stress gradient at the interface region and the defect nucleation were delayed. Sara Fazeli [18] et al. studied the effect of Cu content on the tensile mechanical properties of ternary NiTiCu alloy nanowires and the results showed that with the increase of copper content, the yield strength and Young’s modulus decrease. Shen [19] carried out molecular dynamics simulation of nanopolycrystalline copper under different strain rates and observed that the yield strength and ductility of nanopolycrystalline copper under uniaxial stress loading increased with the increase of strain rate. According to Li’s [20] work, the elastic modulus increases firstly and then stabilizes as the strain rate increases, and the flow strength increases with the increase of strain rate. Chang [21] et al. carried out molecular dynamics simulation of Ti nanowires, and the results showed that when the strain rate was higher than 0.01 ps^−1^, the tensile properties of the nanowires improved rapidly, and the yield point and yield stress both increased with the increase of strain rate. Zhang [22] et al. studied the effect of strain rate on the plastic deformation of nanopolycrystalline copper and found that higher strain rate would lead to stacking fault intersection, thus increasing the flow stress. Therefore, it is feasible to study the mechanical properties of nanopolycrystalline alloys through molecular dynamics simulation and may also be suitable for nanopolycrystalline Cu–Sn alloys.

Therefore, this study explored the influencing factors of mechanical properties of nanopolycrystalline Cu–Sn alloy by the molecular dynamics method. The effects of Sn element proportion and strain rate on the mechanical properties of nanopolycrystalline Cu–Sn alloy were studied systematically. Relative mechanisms of high strength, elastic modulus and good ductility of nanopolycrystalline Cu–Sn alloys were revealed. This study may provide an important reference and feasible method for the research of nanopolycrystalline Cu–Sn alloy and promote the development and application of Cu–Sn alloys.

## 2. Simulation Methods

**Figure 1:**
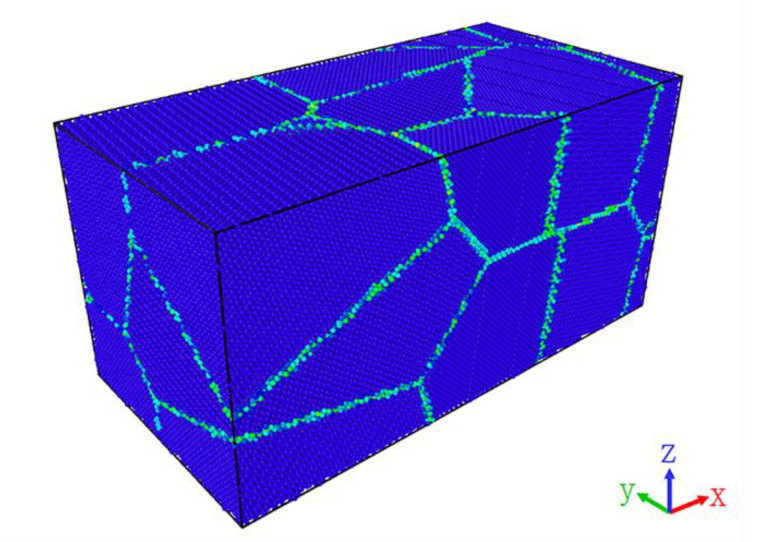
The model of nanopolycrystalline Cu–Sn alloy.

The present study researched the deformation behavior of nanopolycrystalline Cu–Sn alloy during the tensile process, and the Voronoi method [23] was used to establish nanopolycrystalline Cu–Sn alloy models as shown in Figure 1. The periodic boundary conditions were applied in the X, Y and Z directions of the polycrystalline model. According to the research needs, ten models with different proportions of Sn elements which were 2%, 3%, 4%, 5%, 6%, 7%, 8%, 9%, 10% and 11% were established, respectively. The method of random substitution of atoms was utilized to build the model. Firstly, the polycrystalline Cu model was established, then Cu atoms were randomly replaced by different proportions of Sn atoms. The average grain size of the model was 6.92 nm, the number of atoms in all models is 569,133. The analysis and visualization process were carried out using the software OVITO [24].

In the present study, the uniaxial (X-direction) tensile simulation of Cu–Sn alloy was carried out. The energy minimization and relaxation were performed before stretching, and the conjugate gradient algorithm was used to achieve minimum energy and stable structures. Then, the load was applied to the model with different Sn element ratios, and the tensile simulation was performed under the isothermal–isobaric ensemble (NPT). The load was evenly applied on the *x*-axis. The time step in the stretching process was set as 0.001 ps, the temperature of the system was set as 300 K, and the strain rate was 5 × 10^9^ s^−1^.

Tensile simulations were performed using the Large-scale Atomic/Molecular Massively Parallel Simulator (LAMMPS) [25] which was developed by Sandia National Laboratories. The more mature modified embedded-atom method (MEAM) [26] potential was used to describe the interactions of Cu and Sn. In the MEAM formulation, the total energy *E* of a system of atoms is shown in Equation (1):

(1) $$E\;=\;\sum_{i}\;\{F_{i}\left(\bar{\rho_{i}}\right)\;+\;\frac{1}{2}\;\sum_{i\neq j}\phi_{\mathrm{ij}}\;\left(r_{\mathrm{ij}}\right)\}$$

where *F* is the embedding energy which is a function of the atomic electron density *ρ*, *ϕ_i__j_* is a pair potential interaction and *r_ij_* is the distance between atoms *I* and *J*.

## 3. Results and Discussion

### 3.1. Effect of Sn Content on Properties of Nanopolycrystalline Cu–Sn Alloy

**Figure 2:**
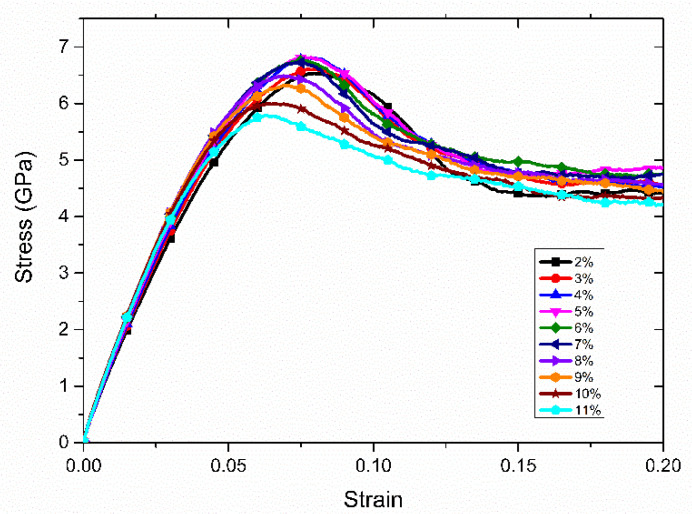
The stress–strain curves obtained by stretching Cu–Sn alloy models with different Sn content.

The stress–strain curves of Cu–Sn alloy with different Sn content obtained by tensile simulation are presented in Figure 2. It can be seen that all of the alloy systems with different Sn contents undergo the same deformation stage under tensile load, which are the elastic deformation stage and plastic deformation stage, respectively. The tensile deformation begins with elastic deformation, and the stress–strain relationship changes linearly. It is obvious that the elastic modulus of the alloy increases firstly, and then decreases with the increase of the proportion of Sn element.

After the elastic deformation stage, the alloy system enters the plastic deformation stage with the loading continuing. According to the stress–strain curve, the stress reaches the peak value, then decreases. By comparing the highest point of the stress–strain curve, it can also be observed that the tensile strength of the alloy system increases firstly and then decreases with the increase of Sn content. At the same time, with the increase of Sn content, the strain corresponding to the tensile strength of different systems is decreasing. This indicates that the ductility of Cu–Sn alloy is weakened with the addition of the Sn element. By fitting the slope of the elastic stage curve, the elastic modulus corresponding to different Sn contents can be obtained.

**Figure 3:**
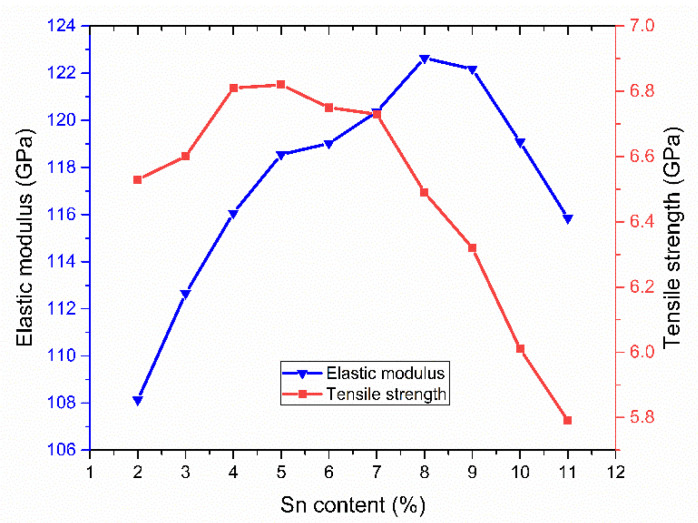
The elastic modulus and tensile strength as a function of Sn content.

The elastic modulus and tensile strength under different Sn content were plotted, as shown in Figure 3. It can be concluded that both the elastic modulus and yield strength curve showed a trend of firstly increasing and then decreasing. The elastic modulus increases from 108.13 GPa to 122.64 GPa when the content of Sn ranges from 2% to 8%, but it decreases when the Sn contents range from 8% to 11%. The tensile strength increases monotonously with 2–4% Sn contents and decreases when the Sn contents are 4–11%. Furthermore, when the Sn content exceeds 8%, the curve is steeper, and the decrease is more obvious. Compared with the elastic modulus, the tensile strength decreased early. The probable reason may be that the elastic modulus essentially reflects the strength of the binding energy between the atoms of the material, which is closely related to the characteristics of the crystal itself. The higher atomic density enhances the interatomic binding force and increases the elastic modulus.

The tensile strength reflects the strength and plasticity of the material. Sn plays a role of solid solution strengthening for the whole alloy system, which enhances the binding energy between Cu and Sn atoms. Moreover, the atomic radius of an Sn atom is larger than a Cu atom, which leads to the increasing lattice distortion and more obvious strengthening effect. Therefore, in a certain range, with the increasing proportion of the Sn element, the elastic modulus and tensile strength increase. On the other hand, the addition of Sn will reduce the plasticity and toughness of the material. Therefore, when the proportion of Sn increases to a certain amount, the effect of brittleness on the material is greater than strengthening, leading to the elastic modulus and tensile strength of nanopolycrystalline Cu–Sn alloy starting to decline. By observing the inflection point in Figure 3, it is known that the tensile strength is more sensitive to the addition of the Sn element, indicating that when the Sn element is too much, the nanopolycrystalline Cu–Sn alloy is more prone to brittle fracture, which seriously affects the mechanical properties of the material.

**Figure 4:**
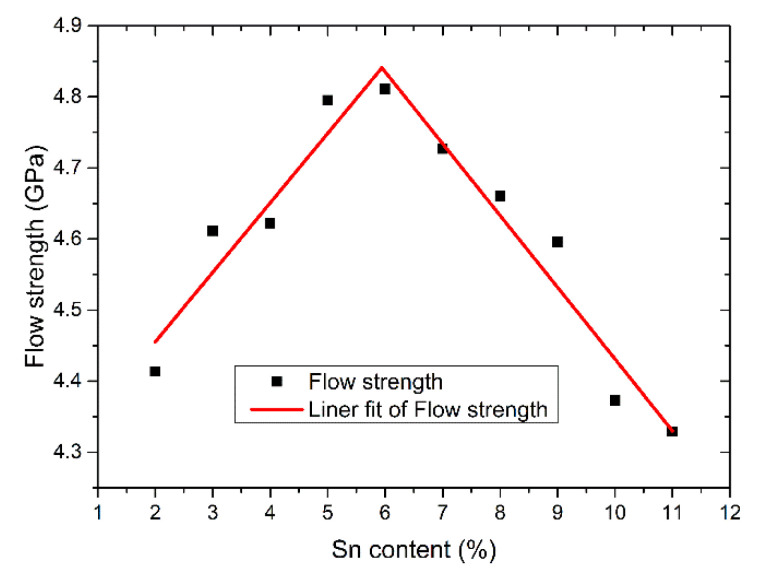
The flow strength as a function of Sn content.

Generally, the flow strength describes the strength of the plastic deformation of materials. The flow strengths are obtained by fitting the average value of the stress between the strain of 0.15 and the strain of 0.2, as shown in Figure 4. The effect of Sn content on flow strength has the same trend as elastic modulus and tensile strength. It can be seen from the fitting curve of flow strength that when the Sn content is between 2% and 6%, the flow strength increases with the increase of the Sn content. When the Sn content is 6%, the curve changes suddenly. When the Sn content is between 6% and 11%, the flow strength decreases with the increase of Sn content. Sn content also has a significant effect on flow strength.

### 3.2. Effect of Strain Rate on Properties of Nanopolycrystalline Cu–Sn Alloy

**Figure 5:**
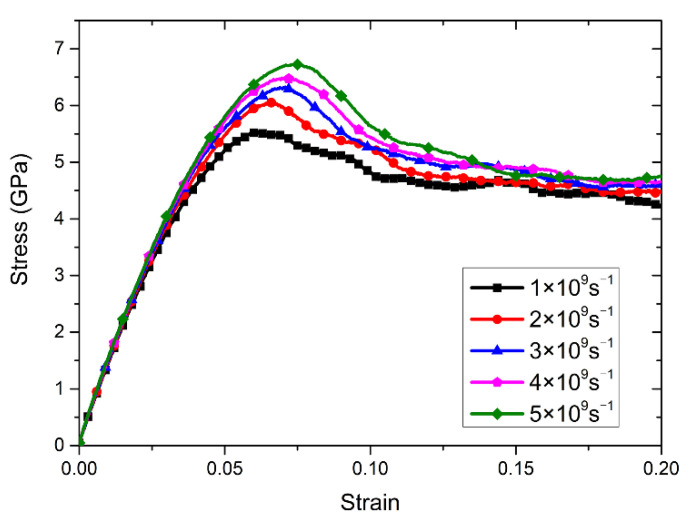
The stress–strain curves obtained by stretching Cu–7Sn alloy models with different strain rates.

The mechanical properties and deformation behavior of materials are also associated to the strain rate. The effect of strain rate on mechanical properties of nanopolycrystalline Cu–Sn alloy was studied. From the previous section, the mechanical properties are better when the Sn content is 7%. In this section, strain rates of 1 × 10^9^ s^−1^, 2 × 10^9^ s^−1^, 3 × 10^9^ s^−1^, 4×10^9^ s^−1^ and 5 × 10^9^ s^−1^ were applied to the Cu–Sn alloy model with an average grain size of 6.92 nm and Sn content of 7%, respectively. The stress–strain curves obtained by tensile calculation were shown in Figure 5.

It can be concluded from Figure 5 that the tensile process also experienced elastic deformation and plastic deformation. In the elastic deformation stage, the slope of the stress–strain curve increases slowly with the increase of the strain rate, which indicates that the strain rate weakly influences the elastic modulus. However, the tensile strength evidently increases with the increase of the strain rate, which indicates that the tensile strength is more susceptible to be influenced by the strain rate. Moreover, the greater the strain rate, the sharper the decrease in the stress–strain curve after reaching the peak, and the curve is steeper. At the same time, as the strain rate increases, there is greater strain when the material reaches the tensile strength.

**Figure 6:**
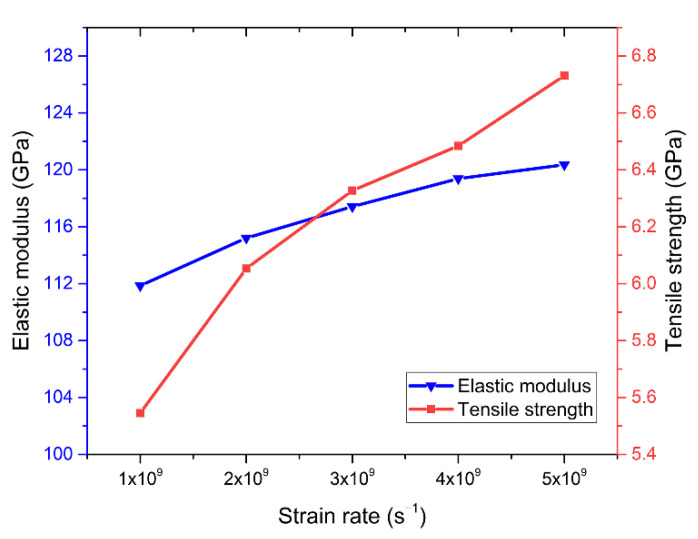
The elastic modulus and tensile strength as a function of strain rate.

Similarly, in order to observe the effect of strain rate on the mechanical properties of Cu–Sn alloy more intuitively, Figure 6 shows the variation of elastic modulus and tensile strength with different strain rates. It can be found that when the strain rate was 1×10^9^/s^−1^, the elastic modulus was 111.86 GPa and the tensile strength was 5.545 GPa. When the strain rate was 5×10^9^/s^−1^, the elastic modulus was 120.36 GPa and the tensile strength increased to 6.731 GPa. The elastic modulus only increased by 7.6%, and the tensile strength increased by 21.4%. At high strain rates, the alloy system reached its peak more slowly during the tensile process, and the ductility of the material was relatively enhanced with the increase of the strain rate. With the increase of strain rate, the elastic modulus curve was relatively flat and the increase was small, while the tensile strength curve was relatively steep and the increase was obvious. Huang et al. also found that the change of strain rate has little effect on the elastic modulus of materials under tension [27]. The above data demonstrate that the strain rate has a weak effect on the elastic modulus, but a greater effect on the tensile strength. Therefore, the higher strain rate has a significant effect on the strengthening of Cu–Sn alloy.

**Figure 7:**
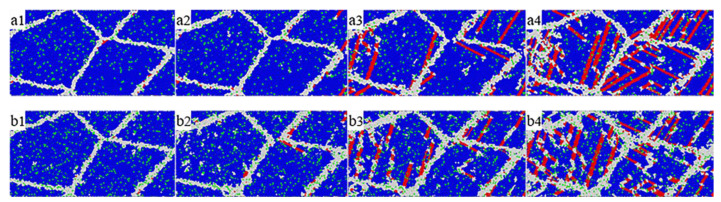
Tensile deformation process of nanopolycrystalline Cu–Sn alloys with different Sn contents. (**a**) Atomic structure with Sn content of 5%; (**a1**) ε = 0.05; (**a2**) ε = 0.0700; (**a3**) ε = 0.1; (**a4**) ε = 0.15. (**b**) Atomic structure with Sn content of 8%; (**b1**) ε = 0.05; (**b2**) ε = 0.0650; (**b3**) ε = 0.1; (**b4**) ε = 0.15.

**Figure 8:**
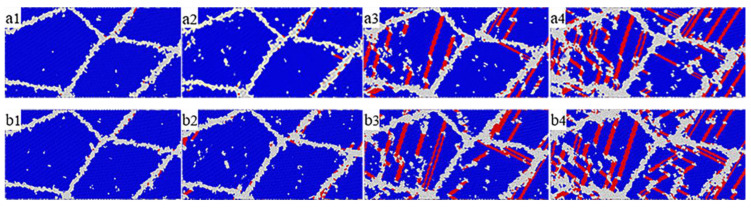
Tensile deformation process of nanopolycrystalline Cu–7Sn alloys with different strain rate. (**a**) Atomic structure with strain rate of 3 × 10^9^ s^−1^; (**a1**) ε = 0.05; **a2**) ε = 0.0675; (**a3**) ε = 0.1; (**a4**) ε = 0.15. (**b**) Atomic structure with strain rate of 5 × 10^9^ s^−1^; (**b1**) ε = 0.05; (**b2**) ε = 0.0650; (**b3**) ε = 0.1; (**b4**) ε = 0.15.

In order to more directly observe the microstructure evolution of nanopolycrystalline Cu–Sn alloy during tensile simulation process, the typical atomic structure diagrams of the alloy system with a Sn content of 5% and 8% and strain rate of 3 × 10^9^ s^−1^ and 5 × 10^9^ s^−1^ are shown in Figure 7 and Figure 8. Common neighbor analysis (CNA) [28] method was used to analyze the changes of atoms under tensile load to distinguish the grain boundary atoms and dislocations produced during the tensile process. The atomic structures were distinguished by different colors: blue represents face-centered cubic (FCC) structure, red represents hexagonal close-packed (HCP) structure, green represents the Sn atom in the system, and white represents OTHER atomic structures. In OVITO software, FCC structure is used to represent the perfect lattice structure, HCP structure is used to represent the dislocations and defects in the tensile process, and OTHER structure is used to represent the disordered atomic structure. Grain boundaries were defined as OTHER atomic structures, which were represented by white atoms in the figure.

From Figure 7, it can be seen that the alloy system with 5% Sn and 8% Sn was in the elastic deformation stage when the strain was between 0 and 0.07 and 0 and 0.065, respectively. This can be verified by the stress–strain curve as shown in Figure 2. Moreover, by observing the atomic structure diagram of ε = 0.05, the grain boundary changes slightly. The nanopolycrystalline Cu–Sn alloy only underwent elastic deformation. When the strain was at ε = 0.07 and ε = 0.065, respectively, some FCC lattice structure changes to HCP lattice structure and partial dislocation began to appear at the grain boundary, as well as the gradual extension of the grain interior, which indicates that the alloy system reaches the yield point and begins to enter the plastic deformation stage. In the tensile process, the dislocation is the result of the irregular movement of atoms, which destroys the original atomic structure and generates new structural types. In FCC structure metals, tensile load makes the atoms move continuously, the original structure is destroyed and dislocations are generated, the phase transformation occurs in the material and FCC structure changes to HCP structure [29]. Moreover, from Figure 7, we observed the generation of twins, which is the result of the cross-slip of the HCP structure during the stretching process. Dislocations make it easier for atoms to move with each other, and when the same strain is generated, the required stress is smaller, so the stress drops in the stress–strain curve.

**Figure 9:**
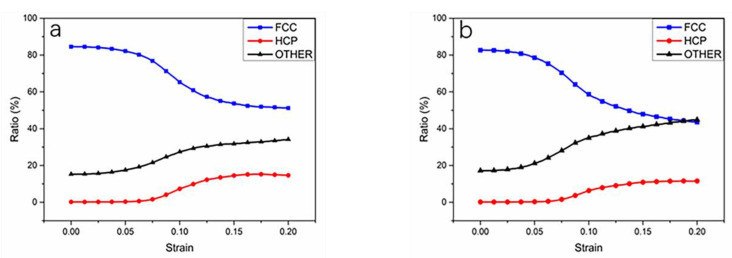
Lattice ratio–strain curve for Cu–Sn alloy models with Sn contents of (**a**) 5% and (**b**) 8%.

Through comparison, it is obvious that the alloy system with 8% Sn content reaches the yield point earlier than the alloy system with 5% content, and the system with 8% content has dislocations not only emitted from the grain boundaries, but also inside the grains, indicating that the increase in Sn atom content has changed the dislocation generation mechanism of the alloy system. With the increase of strain, some grain boundaries are fractured and recombined, and some dislocations are generated from one side of the grain boundary, grow up and penetrate the whole grain, and then annihilate on the other side of the grain boundary. Sainath et al. observed a similar phenomenon in their study [30]. In this process, a large number of slip bands are generated, and the proportion of HCP lattice structure is also significantly increased. As shown in Figure 9, the ratios of the FCC, HCP and OTHER structures are calculated. As the strain increases, there is a decrease in the proportion of the FCC lattices as well as an increase in that of the HCP and OTHER lattices for both models. In addition, by comparing with Figure 2, the range where the ratio of lattice changes rapidly and the range where alloy system failure occurs are coincident. This proves that the mechanical properties of nanopolycrystalline Cu–Sn alloy are closely related to the changes in the lattices.

Due to the movement and growth of partial dislocation, stacking faults appear in the grains, and the dislocation density at the trigeminal grain boundary is relatively higher. The continuous generation, expansion and annihilation of dislocations lead to the continuous change of stress in the second half of the tensile process, which leads to a small range of fluctuation in the stress–strain curve. On the other hand, the addition of Sn makes the lattice structure distorted, which hinders the growth and extension of dislocations to a certain extent. As a result, the continuity of stacking faults in the microscopic structure with 8% Sn is worse, and the dislocation density is lower than that in a system with 5% Sn.

**Figure 10:**
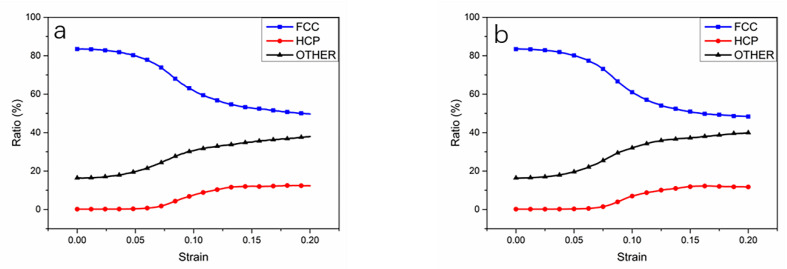
Lattice ratio–strain curve for Cu–Sn alloy models with strain rates of (**a**) 3 × 10^9^ s^−1^ and (**b**) 5 × 10^9^ s^−1^.

Figure 8 shows the atomic structure diagram of tensile deformation of nanopolycrystalline Cu–Sn alloy at strain rates of 3 × 10^9^ s^−1^ and 5 × 10^9^ s^−1^. Cu–Sn alloy is in the elastic deformation stage when the strain was between 0 an 0.0675 and 0 an 0.065, respectively. The a3–b3 and a4–b4 in Figure 8, show that the increase of strain rate is conducive to the formation of stacking faults in the Cu–Sn alloy. Figure 10 shows the atomic ratio with strain of 3 × 10^9^ s^−1^ and 5 × 10^9^ s^−1^. It can be observed that when both models have the same strain, the internal dislocation density of Cu–Sn alloy is larger at a high strain rate. In addition, the accumulation of stacking faults will hinder the extension of dislocations and require greater force when the same strain is generated, which shows the strength of the material is strengthened. Combined with the microscopic structure, the increase of strain rate can improve the tensile strength of the material, so that the strength of the material increases, and the ductility of the material also improves.

## 4. Conclusions

In this work, the mechanical properties of nanopolycrystalline Cu–Sn alloy were analyzed using a molecular dynamics method. Firstly, ten alloy models with different Sn element contents were established, and tensile simulations were performed on them. Then, different strain rates were applied to the Cu–7Sn alloy model for tensile simulation. Finally, microstructure evolution of nanopolycrystalline Cu–Sn alloys under different content and different strain rates was analyzed. The results can be summarized as follows:

(1) Sn content has a significant effect on the mechanical properties of nanopolycrystalline Cu–Sn alloy. The elastic modulus and tensile strength of the material depend on the proportion of Sn in the alloy. The results demonstrate that the addition of Sn reduces the ductility of nanopolycrystalline Cu–Sn alloy. However, the elastic modulus and tensile strength of nanopolycrystalline Cu–Sn alloy are improved by increasing the Sn content initially, but they will be reduced when the Sn content exceeds 4% and 8%, respectively.
(2) The strain rate weakly influenced the elastic modulus of nanopolycrystalline Cu–7Sn alloy, but the tensile strength and ductility are obviously enhanced with an increasing strain rate. The tensile strength increases with the increase of the strain rate, and the strain rate has a significant strengthening effect on the material.

## Data Availability

Data sharing is not applicable for this article.
